# Supplementary material for: Analysis of Dynamic Plantar Pressure and Influence of Clinical-Functional Measures on Their Performance in Subjects with Bimalleolar Ankle Fracture at 6 and 12 Months Post-Surgery
Source: Sensors (Basel). 2023 Apr 13;23(8):3975. doi: 10.3390/s23083975 (PMC10142754; doi:10.3390/s23083975)
Supplement: Supplementary file 1 [file sensors-23-03975-s001.zip › sensors-2267022-supplementary.pdf]

Table S1. Multiple linear regression models for dynamic plantar pressure variables. AFG at 6 months post-surgery.

**Models.**	***F***	***R^2***	***ΔR^2***	***Constant***<br>***B***	***Standard Error***	***β***	*1- β*	*f^2*
**Contact time**				1923.35	147.81			
Cadence	34.5 (1.21)	0.79	0.76	−6.70	2.01	−0.49 *	0.99	3.7
Speed				−450.34	137.26	−0.48 *		
**Peak plantar pressure**				3.09				
Bimalleolar perimeter	9.9 (1.21)	0.33	0.30	−0.07	0.02	−0.58 **	0.83	0.43
**Average plantar pressure**				1.58				
Bimalleolar perimeter	7.5 (1.21)	0.27	0.23	−0.03	0.01	−0.52 *	0.68	0.30

*$p < 0.05$; ** $p < 0.001$; F= ANOVA; R^2 = Coefficient of determination; ΔR^2 = *R* Square adjusted; *B*: Non-standardised coefficient; *β* = Standardised coefficient; *1- β*: Statistical power; f^2 = Effect size.

Table S2. Multiple linear regression models for dynamic plantar pressure variables. AFG at 12 months post-surgery.

**Models**	***F***	***R^2***	***ΔR^2***	***Constant***<br>***B***	***Standard Error***	***β***	***1- β***	***f^2***
**Contact time**				1612.91	147.81			
Cadence	21.3 (1.20)	0.70	0.67	−5.66	1.44	−0.55 **	0.99	2.1
Speed				−246.61	64.74	−0.51 **		
**Peak plantar pressure**				3.38				
Bimalleolar perimeter	15.8 (1.20)	0.45	0.43	−0.08	0.02	−0.67 **	0.96	0.75
**Average plantar pressure**				1.78				
Bimalleolar perimeter	9.9 (1.20)	0.34	0.30	−0.43	0.01	−0.59 *	0.81	0.42

*$p < 0.05$; ** $p < 0.001$; F= ANOVA; R^2= Coefficient of determination; ΔR^2= *R* Square adjusted; *B*: Non-standardised coefficient; *β*= Standardised coefficient; *1- β*: Statistical power; f^2= Effect size.
